# Emulsifiers: Their Influence on the Rheological and Texture Properties in an Industrial Chocolate

**DOI:** 10.3390/molecules29215185

**Published:** 2024-11-02

**Authors:** Maria Pombal, Ismael Marcet, Manuel Rendueles, Mario Diaz

**Affiliations:** 1 Department of Chemical and Environmental Engineering, University of Oviedo, C/Julián Clavería s/n, 33071 Oviedo, Spain; mariapombal86@gmail.com (M.P.); marcetismael@uniovi.es (I.M.); mariodiaz@uniovi.es (M.D.); 2 Chocolates Lacasa, 33199 Siero, Spain

**Keywords:** chocolate, emulsifiers, rheological properties, thixotropic behaviour, textural properties

## Abstract

The complexity of the chocolate matrix leads to it having characteristic rheological properties that may pose difficulties for its industrial manufacture. Many factors influence the flow behaviour of chocolates, such as raw materials, the amount of fat, the moisture content, particle-size distribution, the concentration of emulsifiers, or manufacturing conditions, among others. This study focusses on the rheological properties of an industrially manufactured chocolate with a 48% cocoa content, and the effect caused by the addition of two emulsifiers (soya lecithin and polyglycerol polyricinoleate (PGPR)) on the rheological properties. In the case of lecithin, a clear effect has been observed on the plastic viscosity and the yield stress. Plastic viscosity decreases until a concentration of 0.6% lecithin is reached, and thereafter remains relatively constant, while yield stress increases over the studied range. This effect is not observed when PGPR is used as the emulsifying agent. In this case, a small concentration of PGPR decreases the yield stress. Thixotropy was determined using the Casson model, and its behaviour was found to be similar to that of plastic viscosity with respect to changes in the PGPR and lecithin concentrations. Textural determinations were also carried out, relating the rheology characteristics to the texturometry.

## 1. Introduction

Chocolate is a product that is widely consumed by all generations, often in large quantities. This is due to its unique aroma and taste, characteristics that make it an object of temptation [1]. Chocolate production, which is a complex process that has improved and developed in recent years, involves numerous chemical and physical reactions that give chocolate its organoleptic and textural properties [2]. Most of these properties perceived by the consumer are related to rheology. In the chocolate industry, the characterisation and control of rheological properties are increasingly important to satisfy consumer standards [3] and determine the machinability of the products [4], due to the influence these rheological properties have on the sensorial characteristics of the final product, namely, creaminess, hardness, and mouthfeel [5]. Moreover, flow parameters play an important role during the solidification of chocolate, as an incompatible flow behaviour might cause poor final product quality [6]. The industry has to adapt the rheology of the chocolate it manufactures to the different end products, especially in the case of covertures. By managing the rheology of the chocolate, it can be used for covertures, shells, or chips and it can also be used with a variety of fillings and for more recent applications such as 3D printing [6]. In fact, the best adapted formulation for a given factory process can be created [7]. In addition, rheological behaviour is important in industry to determine the energetic requirements of the process, since if the chocolate paste has a high viscosity, it has poorer flow characteristics, and more energy is expended to pump the paste within the factory. Approximately, for the same amount of product with the same pump, a chocolate paste with twice the viscosity takes twice the time to be pumped a given distance because, according to the mathematical definition of viscosity, it is directly proportional to time [8].

In the chocolate manufacturing process, there are four fundamental stages: mixing, refining, conching, and tempering, and all of them influence the rheological properties of the chocolate. But the stages that have the most importance regarding rheological properties are refining and conching. The refining stage is key to rheological behaviour because particle size has an important influence on these properties, and also, the fineness of the chocolate is very important to its organoleptic properties. The size of the particles must be less than 25 µm so that they are not perceptible in the mouth and allow for a good melting of the chocolate during consumption. In the conching stage, a series of physical and chemical processes take place. The solid particles, which behave as a dispersed phase, are coated with butter and acquire a uniform shape. This is related to the rheological and textural properties of the final product. In this step, the rest of the ingredients that were not added in the initial mixing phase are added, such as emulsifiers and the rest of the cocoa butter. Conching is a highly complex process which describes the constant stirring, kneading, and ventilation of the cocoa mass under increased process temperature. It can be divided into three steps: dry, plastic, and liquid. The liquid phase is when the remaining cocoa butter and emulsifiers are added [9]. During conching, it is important to control the working conditions for the development of organoleptic characteristics [8]. In this conching process, flakes of sugar and cocoa liquor are refined, the particles becoming smaller and more rounded, de-agglomeration of the solid particles enclosing the cocoa butter takes place [9], moisture content is reduced, and unpleasant flavours of volatile components related to the origin and characteristics of cocoa are eliminated [8].

Although commercial chocolate can be found in different presentations, normally in solid form, the study of rheological properties focusses on chocolate in a liquid state. Molten chocolate is a suspension of particles of sugar, cocoa, and/or milk solids in a continuous fat phase. The complexity of the microstructure varies depending on the chocolate type and the number and properties of the components. For example, dark chocolate has a simpler microstructure than others, such as milk chocolate [10]. Because of the presence of solid particles in the melted state, chocolate exhibits non-Newtonian flow, which means that the viscosity changes with shear stress [11]. This behaviour is characterised by a yield stress and plastic viscosity. The yield stress is the amount of energy necessary to initiate flow, while the energy to maintain flow is expressed by the plastic viscosity [12]. Another flow behaviour parameter obtained from the rheological analysis is thixotropy. The effect of thixotropic behaviour is the continuous decrease in viscosity when a constant shear rate is applied [13].

The rheological behaviour of liquid chocolate is not only influenced by processing (refining, conching, tempering), as was previously indicated, but also by fat type and ratio, particle-size distribution, composition, concentration of emulsifiers, moisture content, and fat crystal type [4,12]. The influence of the different stages of industrial chocolate production is key to obtaining a final product characterised by a correct texture and appearance [11].

Emulsifiers help to control flow properties. The most frequently used emulsifiers in the chocolate industry are soy lecithin and polyglycerol polyricinoleate (PGPR). These ingredients lower the interfacial tension between the dispersed and the continuous phase of chocolate [4]. Commercial soy lecithin is a complex mixture containing 65–75% phospholipids along with triglycerides and smaller amounts of other substances. The major phospholipids include phosphatidylcholine, phosphatidylethanolamine, and inositol-containing phosphatides. Other reported substances include carbohydrates, pigments, sterols, and sterol glycosides [14]. Lecithin is one of the best emulsifiers for viscosity control, as it reduces production costs by decreasing the amount of cocoa butter needed to achieve similar flow results [15]. Because the lecithin molecules are amphiphilic, they surround the sugar particles, which adsorb their hydrophilic part, while the lipophilic end remains in the fat, thus separating the individual sugar particles. The fat molecules attracted by the lipophilic tails of the lecithin prevent the formation of sugar aggregates but are no longer available to contribute to flow properties [8].

Polyglycerol polyricinoleate, PGPR, is manufactured from polymerized glycerol and polymerized ricinoleic acid and it acts like lecithin. Adequate flow properties can be achieved by the addition of PGPR, which improves the flow characteristics of molten chocolate by reducing yield stress. By contrast, lecithin tends to be used to decrease plastic viscosity [16].

The rheological properties of chocolate are typically examined in its molten state using a viscometer or rheometer. Flow curves are obtained by subjecting the chocolate to a shear stress with a defined geometry and temperature. Flow curves relate viscosity or shear stress to strain rate [6]. There are several mathematical models to describe the rheological behaviour of a non-Newtonian fluid, such as the Ostwald-de Waele, Bingham, Herschel–Bulkley, Casson, and Cross models, among others [17]. In the case of chocolate, the most frequently used is the Casson rheological model, which will be the one employed in this study.

The new lines of research on chocolate attempt to partially replace the content of cocoa butter with, for example, PGPR [18] or a butter–water emulsion [19]. The objectives of these changes are to reduce the price of the finished product, especially in view of recent increases in the cost of cocoa butter, and to obtain a chocolate with a lower amount of fat.

The aim of this study is the characterisation of the rheological parameters and the determination of the thixotropic and textural properties of industrially manufactured 48% cocoa dark chocolate, with respect to the concentrations of two different emulsifiers: lecithin and PGPR. Chocolate with a cocoa content of 48% was chosen as it is one of the most frequently consumed bitter chocolates in the world and the type of chocolate most often used in the industry to be enrobing products, for example, enrobed fruits or wafers. The emulsifiers used were lecithin and PGPR because they are the most used in the chocolate industry and are part of the formula of the chocolate studied. It is therefore very important to determine its rheological properties and how the different emulsifiers influence the rheology of the product, to obtain control over the rheological properties and optimise the process.

## 2. Results and Discussion

### 2.1. Sample Characterisation

The physicochemical properties of the chocolate samples are shown in Table 1, where F1, F2, and F3 are three different batches from an industrial production of dark chocolate with 48% cocoa. The moisture content hardly varied between the different batches. Molten chocolate typically has moisture contents of 0.5–1.5%, mainly present in the cocoa solids, and so not affecting chocolate flow [20].

Most chocolates contain 25–35% fat and the samples measured are in this range. The true level of fat present will depend on the process being used and formulation, and this affects the texture of the finished chocolate [21]. As the fat content increases, the distance between the solid particles increases, so the viscosity decreases [8].

**Figure 1:**
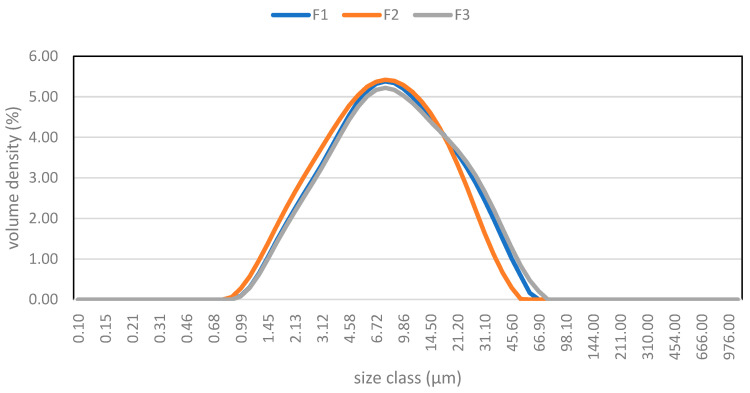
Particle-size distribution of batches F1, F2, and F3.

For the particle-size distribution analysis, the three batches have similar particle distribution curves, but with significant differences (Figure 1), and the results are shown in Table 1. The reported data are the distributions below 90% (D_V90_), below the median (D_V50_), and below 10% (D_V10_). These values indicate that 90%, 50%, or 10% of the volume of the particles has that size or lower. Also displayed is the volumetric area mean (D_(4.3)_).

Therefore, F2 presents a smaller particle size, while in F3, the particle size is larger than in the others. Particle-size distribution has an important effect on the flow properties of chocolates, with a direct influence on sensory perception. If the particles are small, their contact surface is large and, therefore, there is less free fat and the distance between the particles is smaller so the chocolate will be less fluid than if the particles are larger. In the case of F3, the contact surface is small; there is more free fat, the distance between particles is greater, and the chocolate flows more easily [8]. According to Rostagno [22], the F3 batch can be considered as coarse chocolate. This is considered to be chocolate in which more than 20% of the particles exceed the size of 20 µm and are a negative factor for the organoleptic characteristics of the chocolate, as when the chocolate melts in the mouth, the particles produce an unpleasant sensation.

### 2.2. Flow Behaviour

**Figure 2:**
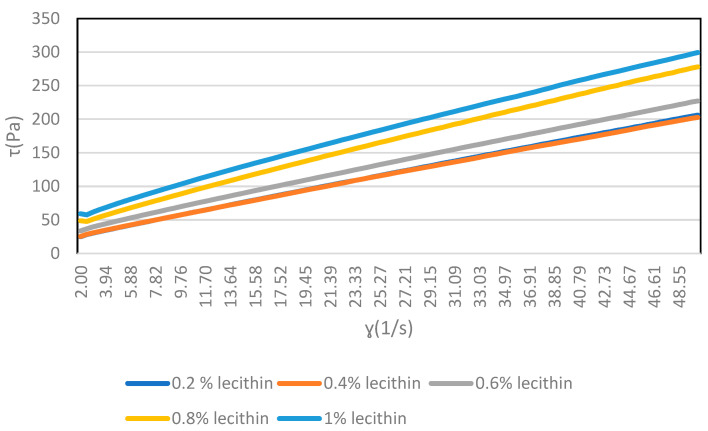
Yield stress versus shear rate from 2 to 50 s^−1^ (ramp up) and the amount of lecithin present for F1.

The base formula (BF) contains 0.3% of lecithin and 0.2% of PGPR, as was indicated in the experimental section. In this study, the amount of lecithin or PGPR was increased by small amounts to determine how these increments affect the rheological properties of the 48% chocolate. Casson’s parameters (yield stress, τ0 and plastic viscosity, ηp) were calculated from the experimental data measured in the rheometer based on Casson’s rheological model. Figure 2 shows an example of experimental data for F1, adding lecithin to the base formula (BF). The figure shows yield stress versus shear rate from 2 to 50 s^−1^ (ramp up). The results of the rheological experiments are shown in Table 2 and Table 3. Table 2 shows the results of modifying the amount of lecithin and Table 3 those of modifying the amount of PGPR. Table 2 and Table 3 also indicate the parameters obtained by fixing the flow curves to the Casson model for the addition of lecithin and PGPR, respectively.

#### 2.2.1. Plastic Viscosity

Table 2 shows the relationship between the Casson viscosity and the amount of lecithin present for F1, F2, and F3 batches.

Table 2 shows that small differences in the initial viscosity of the BF can be observed between the three samples. Rostagno [8,9,10,11,12,13,14,15,16,17,18,19,20,21,22] showed that if the fat content is the same, the viscosity is higher when the particle size is lower. This is because the cocoa butter covers the surface of the particles and if particles are smaller, the contact surface with the cocoa butter is greater. As consequence, the amount of free fat is reduced and the distance between particles is also reduced, which results in an increase in viscosity. For this reason, F1 has lower viscosity than F2, as F1 and F2 have fat contents of 30.57% and 30.62%, respectively, while the volume below 20 µm is 80.8% for F1 and 86.22% for F2 (Table 1). F3 has a higher viscosity than the other two batches, which could be due to it having lower fat content as well as slightly higher moisture content than the other batch. Greater quantities of moisture in the chocolate significantly increase the viscosity. This could be due to the formation of layers of syrup on the surface of the sugar particles, which will increase the friction between them [23].

Lecithin has been shown to be particularly effective in reducing the viscosity of some dark chocolates [24]. It can also be seen in Table 2 that the viscosity varies with the increase in lecithin concentration. The viscosity decreased up to a concentration of approximately 0.5–0.6%, and above that concentration, its behaviour tended to stabilise. In batch 3 (F3), the decrease in viscosity is more pronounced than in F1 and F2, which is a consequence of the fact that the changes lecithin produces are greater when the fat content is lower [13,14,15,16,17,18,19,20,21,22,23,24,25]. In the case of the evolution of the viscosity when increasing the concentration of PGPR, Table 3 shows it was not possible to determine the viscosity value experimentally at higher PGPR concentrations. At these PGPR levels, the Casson parameter(τ_0_) approaches zero, and in these conditions, the rheometer does not give correct results, so the Casson parameters cannot be determined correctly.

#### 2.2.2. Yield Stress

In the case of yield stress (Table 2), differences are also observed between F1, F2, and F3 with respect to the base formula. Yield stress increased more than the viscosity when the chocolate had a large particle size.

When the particle size is reduced, the distance between particles decreases because there are more particles in the same volume and contact friction between them increases, increasing the yield stress [22,23,24,25,26]. In Table 2 and Table 3, it can be observed that F3 has a lower yield stress and has 78.68% particles below 20 µm, while F2 has a higher yield stress and has 86.22% particles below 20 µm.

Relating the physical and chemical properties and the yield stress and viscosity, it can be indicated that the effect of fat is, proportionately, higher on plastic viscosity than on the yield stress value. This effect is due to the fact that a proportion of the fat covers the particles, and the remaining fat molecules move freely, which facilitates the mobility of the cocoa particles, causing an important lubrication effect and thus a large decrease in plastic viscosity. The yield stress is mainly related to the interactions between particles, i.e., to the friction between particles and contact surfaces, so it is less affected than is plastic viscosity by the addition of fat [8].

Table 2 shows the evolution of the yield stress as the concentration of lecithin was raised from 0.3% (BF) to 1.1%. It is observed that the yield stress increases linearly in all cases, the increase being more pronounced in the sample with the smallest particle size. By contrast, the viscosity decreased up to a concentration of approximately 0.5–0.6% of lecithin, and above that concentration, its behaviour tended to stabilise. Moreover, when the concentration of PGPR increases, the yield stress decreases drastically due to the nature of this emulsifier, which can reach a τ_0_ Casson parameter value of zero (Table 3), as mentioned above.

### 2.3. Thixotropic Behaviour

The thixotropic index data are shown in Table 4 and Table 5 using the parameters calculated with the Casson model for the addition of lecithin (Table 4) and PGPR (Table 5).

**Figure 3:**
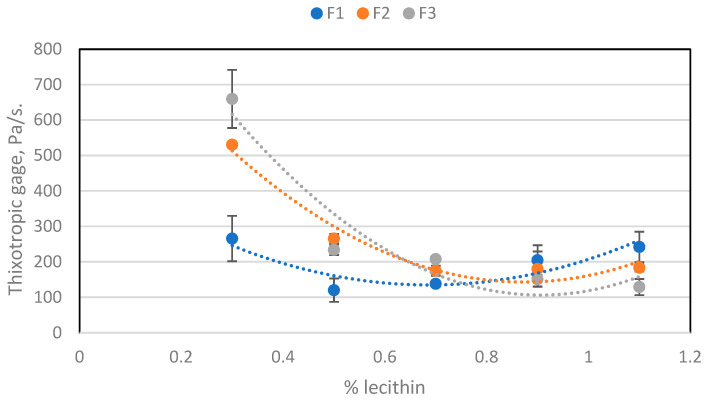
Thixotropic behaviour depending on the amount of lecithin for the three batches.

Figure 3 shows how the thixotropic behaviour of the sample varies with respect to the amount of lecithin. This behaviour undergoes a similar pattern of change as was seen for the viscosity when increasing the amount of lecithin. As already explained in Section 2.2.2, lecithin has a greater effect on viscosity than PGPR. Table 4 shows that there is no significant effect on thixotropic behaviour with the addition of PGPR.

### 2.4. Texture Properties: Stickiness and Hardness

**Figure 4:**
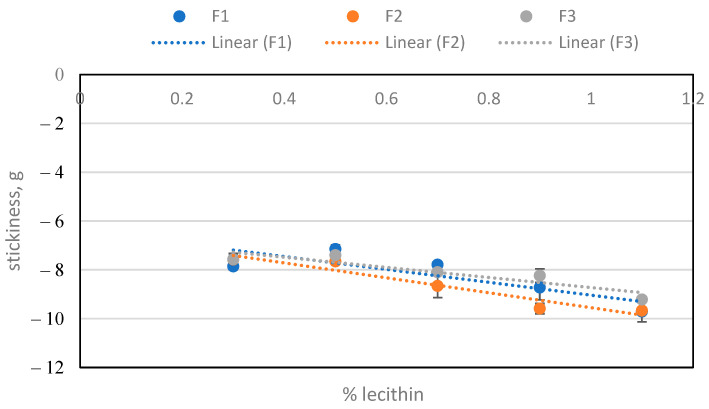
Stickiness depending on the amount of lecithin for the three batches.

**Figure 5:**
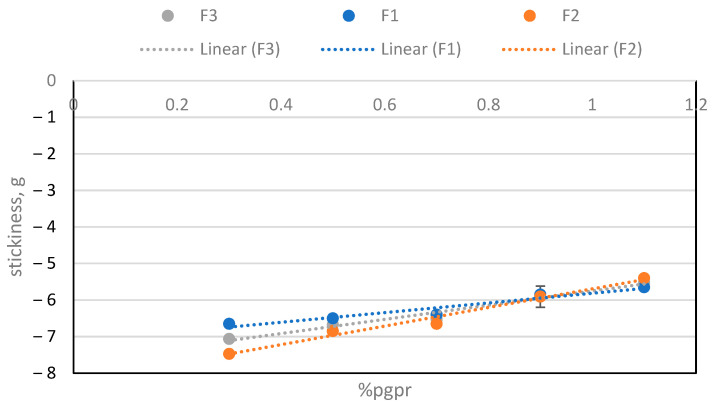
Stickiness depending on the amount of PGPR for the three batches.

The texture properties were measured in the samples after melting at 50 °C. In Figure 4, it can be seen that stickiness increases with lecithin concentration, while Figure 5 shows that stickiness decreases with PGPR concentration. The results obtained have shown significant differences between them, considering a *p* value < 0.05.

**Figure 6:**
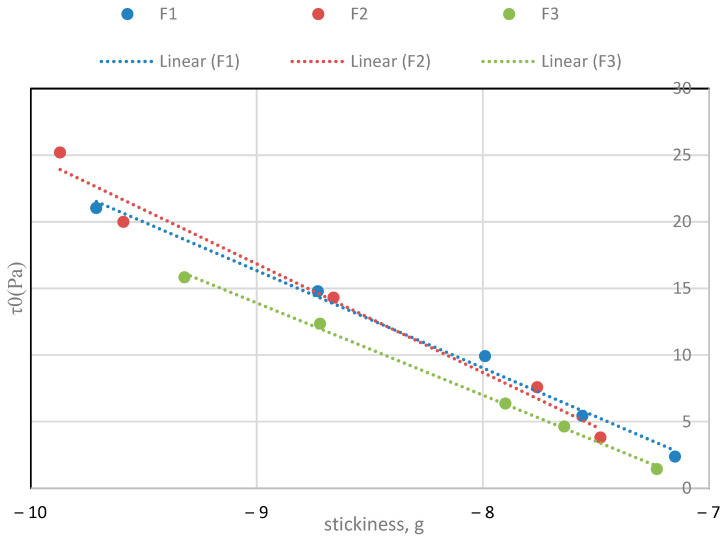
Relationship between stickiness and yield stress with increasing lecithin for the three batches.

This seems to be related to the effect of the lecithin on the yield stress (Table 2). The behaviour of the yield stress, which was derived from the Casson equation (Table 2), and stickiness with increasing lecithin concentration is similar. There is a linear relationship between stickiness and yield stress, with a linear regression of slope of around 7.5 and R^2^ 0.99, as shown in Figure 6.

**Figure 7:**
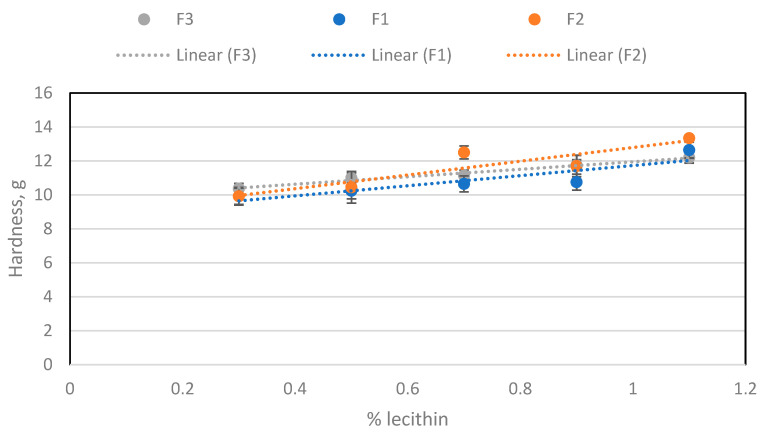
Hardness depending on the amount of lecithin for the three batches.

**Figure 8:**
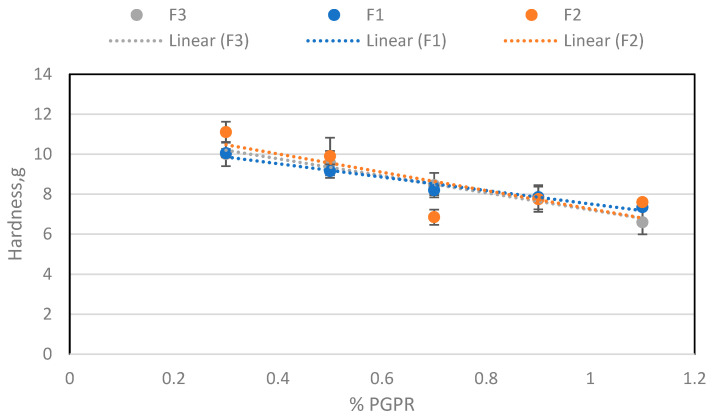
Hardness depending on the amount of PGPR for the three batches.

These data (Figure 6) agree with the perceived experimental sensation of stickiness, when preparing the samples and increasing the amount of lecithin. Therefore, it can be deduced that the cause of this stickiness effect is lecithin. In the case of hardness, its evolution was also studied when adding each emulsifier (Figure 7 and Figure 8). The hardness increased with lecithin concentration and decreased with incremental additions of PGPR to the base formula (BF).

## 3. Materials and Methods

### 3.1. Samples

Samples were taken from an industrial batch of dark chocolate with 48% cocoa. These samples were provided by Chocolates del Norte S.A, from their factory in Asturias, Spain.

Raw materials used in the preparation were cocoa liquor, cocoa butter, sugar, vanillin, and emulsifiers: 0.3% lecithin and 0.2% PGPR. The chocolate is produced on an industrial production line, where raw materials are mixed, ground over 5-roll refiners and conched, using the manufacturers’ production parameters for the recipe.

### 3.2. Preparation of Chocolate Samples

Samples were taken from three different batches, taken at different times of the year, with a time lapse between them of one or two months. The three batches were named as F1, F2, and F3.

The base formula (BF) contains 50–52% sugar, 39–40% cocoa liquor, 9–10% cocoa butter, 0.3% lecithin, and 0.2% PGPR emulsifier. To carry out this study, the total lecithin content was increased, in steps of 0.2%, from 0.3% (BF) until 1.1%. PGPR was also added at concentrations of 0.1, 0.15, 0.2, 0.4, and 0.6%, increasing its total content from 0.2% (BF) to 0.8%.

For analytical purposes, samples were previously melted at 50 °C for 2 h to eliminate crystal memory.

### 3.3. Moisture Content

The moisture content of chocolate was measured using the gravimetric method. Approximately 3–4 g of chocolate was weighed (*M*_0_) and then the sample was heated at 105 °C for 4 h. After that, it was cooled in the desiccator for 30 min and was weighed again to give the final mass of the sample (*M*_1_), which represents only the mass of solid particles with no water. The water content of the chocolate can be calculated using Equation (1). Moisture is one of the main factors influencing the flow behaviour of chocolate [27].

Moisture content (%) = (M_0_ − M_1_)/M_0_ × 100 (1)

### 3.4. Fat Contents

The Soxhlet method was used for the extraction of fat but due to the complexity of the chocolate matrix, a previous extraction was carried out. For this, a mixture of approximately 4 g of sample was digested with distilled water and 0.1M, 25% (*w*/*w*) hydrochloric acid. The mass obtained from filtering this mixture was processed by the Soxhlet method for 6 h, using petroleum ether as solvent.

### 3.5. Particle-Size Distribution (PSD)

To measure the PSD of the chocolates, a MasterSizer 3000E (laser-diffraction particle-size analyser, Malvern Instruments LTD, Malvern) with a Hydro SM was used. Approximately 5 g of melted chocolate was mixed with 100 mL of isopropanol. The sample was sonicated for 5 min and then loaded into the particle analyser until an obscuration of 10% was reached. The size distribution was measured as relative volume of particles [28].

### 3.6. Rheological Analysis

Rheological properties were measured using a Haake Mars II rotational rheometer with a probe and PP60ti plate with a 1 mm gap. Chocolate samples were melted at 50 °C for 2 h and approximately 2 g of the sample was placed onto a preheated plate. The measurement procedure was based on the method of ICA [29] with a minor modification, consisting of a pre-sheared time of 300 s instead of 500 s, as used in the former method. The measurement cycle started to precondition at 40 °C for 300 s. The sample was then pre-sheared at 5 s^−1^ for 300 s. Shear stress was determined with increasing shear rate from 2 to 50 s^−1^ for 180 s (ramp up), then a constant shearing at 50 s^−1^ for 60 s. In the last step, a decrease in the shear rate from 50 to 2 s^−1^ for 180 s (ramp down) was carried out. Each rheological analysis was executed in triplicate.

The most common model used to depict the rheological behaviour of chocolate is the Casson model. The Casson model is a structure-based model defined by Equation (2). The parameter τ (shear stress) is measured, in the rheometer, from the obtained values of time and ɣ (shear rate), as described above. From these values, the Casson parameters ${ɳ}_{p}$ (plastic viscosity), $\tau_{0}$ (yield stress), and exponent n, which in this case is 0.5, can be obtained.

(2) $$\tau =\sqrt[{n}]{\tau_{0}+{\left(ɣ*{ɳ}_{p}\right)}^{n}},$$

where, τ is shear stress (Pa), *ɣ* is shear rate (s^−1^), ${ɳ}_{p}$ is plastic viscosity (cP), $\tau_{0}$ is yield stress (Pa).

Thixotropy is another flow parameter that can be used to evaluate the performance of the chocolate processing method. From the data obtained experimentally in the rheometer, the thixotropic index is calculated by the software of the rheometer by taking the area between the ramp up and ramp down of the flow curves.

### 3.7. Texture Analysis

The texture analysis was carried out with a TA TX plus texture analyser. Samples of approximately 50 mL were measured in the liquid state. For this, chocolate samples were previously melted at 50 °C for 2 h. An SMS P/0.5 sensor with a 30 kg load cell was used. Speed was 2 mm/s and distance 20 mm. The measured parameters were hardness and stickiness. Hardness is the maximum force required to penetrate the sample [30] and stickiness is the force of adhesion between two surfaces [31].

### 3.8. Statistical Analysis

Data were statistically analysed by one-way analysis of variance (ANOVA) using Statgraphics software. This test was used to determine the significant differences at a level of *p* < 0.05. All experimental data were obtained in triplicate.

## 4. Conclusions

In the chocolate industry, the characterisation and control of the rheological properties is very important to satisfy consumer standards, due to the influence of these rheological properties on the sensorial characteristics of the final product. The emulsifiers help to control flow behaviour. This control is very important for the homogeneity of the production in the industrial production of chocolate. The effect of lecithin and PGPR on the rheology of an industrial chocolate containing 48% cocoa, whose base formula (BF) contained 0.3% lecithin and 0.2% PGPR, was determined in this study. The emulsifiers, lecithin and PGPR, are the most commonly used in the chocolate industry. Lecithin is characterised by its effect on viscosity and PGPR is of great interest due to its impact on the yield stress. The range of emulsifier concentrations chosen was that used industrially, for lecithin up to approximately 0.6% and for PGPR up to 0.3%, and higher, in order to observe the behaviour of the rheological properties. Physical and chemical parameters (moisture, fat, and particle-size distribution) differed slightly between the three industrial batches tested, F1, F2, and F3. The results show that emulsifiers such as lecithin and PGPR considerably influence the rheological properties of plastic viscosity and yield stress. Plastic viscosity decreased with increasing lecithin concentration up to a concentration of 0.5–0.6%, and then stabilised despite further addition of lecithin. The yield stress increased with increasing lecithin concentration. The thixotropic character of the chocolate decreased with the addition of lecithin to reach concentrations above that of the base formula, and viscosity behaved in a similar way as lecithin was added. However, no effect on either of these properties could be observed on increasing the concentration of PGPR above that of the base formula. The textural properties of stickiness and hardness increased with lecithin addition and decreased with PGPR addition. This supports the theory that lecithin generates stickiness in chocolate, since it causes a rise in yield stress as its concentration increases. From this, it can be concluded that controlling the rheological parameters in the production process is important to achieve the desired specifications of the finished product (texture, melting, and quality of the chocolate), which is necessary to ensure a continuous flow rate when the chocolate is used for covertures, for instance. The control of chocolate properties such as particle-size distribution, moisture, fat content, and the addition of emulsifiers allows the rheological behaviour of the product to be managed, both to ensure a high-quality product and to reduce processing costs.

## Figures and Tables

**Table 1:** Physical and chemical properties of 48% chocolate industrial batches.

Sample	% Moisture	% Fat	Particle-Size Distribution				
			D_(4.3)_	D_v(10_)	D_v(50)_	D_v(90)_	%Volume Below 20 µm
F1	0.54 ± 0.06	30.57 ± 0.51	12.4	2.72	8.74	28	80.8
F2	0.51 ± 0.01	30.62 ± 0.58	10.5	2.42	7.78	22.9	86.22
F3	0.53 ± 0.04	29.76 ± 0.62	13.3	2.76	9.04	30.3	78.68

**Table 2:** Rheological properties and Casson parameters of 48% chocolate industrial batches F1, F2, and F3, adding lecithin to the base formula (BF).

SAMPLE	% PGPR (Total Quantity)	% Lecithin (Total Quantity)	τ0 (Pa)	ηp (cP)	r
F1	0.2	0.3	2.37 ± 0.76	3.56 ± 0.54	0.9994
	0.2	0.5	5.42 ± 0.28	2.77 ± 0.10	0.9996
	0.2	0.7	9.91 ± 0.27	2.77 ± 0.09	0.9995
	0.2	0.9	14.78 ± 0.90	2.89 ± 0.62	0.9994
	0.2	1.1	21.02 ± 0.12	3.09 ± 0.10	0.9996
F2	0.2	0.3	3.80 ± 0.79	4.67 ± 0.18	0.9987
	0.2	0.5	7.57 ± 0.30	3.52 ± 0.03	0.9993
	0.2	0.7	14.26 ± 0.08	2.91 ± 0.03	0.9993
	0.2	0.9	19.98 ± 0.47	2.8 ± 0.05	0.9995
	0.2	1.1	25.19 ± 0.23	2.68 ± 0.03	0.9995
F3	0.2	0.3	1.43 ± 0.27	4.9 ± 0.21	0.9991
	0.2	0.5	4.62 ± 0.40	4.19 ± 0.03	0.9994
	0.2	0.7	6.35 ± 0.04	3.69 ± 0.06	0.9996
	0.2	0.9	12.34 ± 0.06	3.36 ± 0.15	0.9996
	0.2	1.1	15.82 ± 0.70	3.11 ± 0.10	0.9996

**Table 3:** Rheological properties and Casson parameters of 48% chocolate industrial batches F1, F2, and F3, adding PGPR to the base formula (BF).

SAMPLE	% Lecithin (Total Quantity)	% PGPR (Total Quantity)	τ0 (Pa)	ηp (cP)	r
F1	0.3	0.2	3.71 ± 0.76	4.50 ± 0.54	0.9999
	0.3	0.3	0.47 ± 0.10	4.81 ± 0.30	0.9999
	0.3	0.35	0.16 ± 0.03	4.93 ± 0.21	0.9999
	0.3	0.4	0.03 ± 0.01	3.26 ± 0.19	0.9999
	0.3	0.6	NA		
	0.3	0.8	NA		
F2	0.3	0.2	5.22 ± 0.79	4.34 ± 0.20	0.9999
	0.3	0.3	0.18 ± 0.10	4.99 ± 0.06	0.9999
	0.3	0.4	NA		
	0.3	0.6	NA		
	0.3	0.8	NA		
F3	0.3	0.2	1.89 ± 0.80	4.93 ± 0.21	0.9977
	0.3	0.3	1.62 ± 0.30	5.59 ± 0.15	0.9999
	0.3	0.4	NA		
	0.3	0.6	NA		
	0.3	0.8	NA		

**Table 4:** Thixotropic index variation with the addition of PGPR to the base formula (BF) for 48% chocolate industrial batches F1, F2, and F3.

SAMPLE	% Lecithin (Total Quantity)	% PGPR (Total Quantity)	Thixotropic Index (Pa/s)
F1	0.3	0.2	343.0 ± 21.5
	0.3	0.3	289.8 ± 15.9
	0.3	0.35	274.0 ± 12.3
	0.3	0.4	286.9 ± 10.2
F2	0.3	0.2	527.3 ± 30.2
	0.3	0.3	415.4 ± 39.4
F3	0.3	0.2	514.1 ± 20.5
	0.3	0.3	322.8 ± 13.6

**Table 5:** Thixotropic index variation with the addition of lecithin to the base formula (BF) for 48% chocolate industrial batches F1, F2, and F3.

SAMPLE	% PGPR (Total Quantity)	% Lecithin (Total Quantity)	Thixotropic Index (Pa/s)
F1	0.2	0.3	202.4 ± 40.1
	02	0.5	119.9 ± 32.9
	0.2	0.7	138.0 ± 8.0
	0.2	0.9	204.5 ± 35.6
	0.2	1.1	242.0 ± 24.1
F2	0.2	0.3	534.9 ± 8.3
	0.2	0.5	265.4 ± 13.3
	0.2	0.7	175.0 ± 14.3
	0.2	0.9	180.0 ± 35.6
	0.2	1.1	183.3 ± 10.6
F3	0.2	0.3	641.0 ± 14.8
	0.2	0.5	234.0 ± 15.1
	0.2	0.7	207.8 ± 20.1
	0.2	0.9	150.5 ± 20.3
	0.2	1.1	128.9 ± 22.9

## Data Availability

Data are unavailable due to privacy.

## References

[1] Medina-Mendoza M., Castro-Alayo E.M., Balcazar-Zumaeta C.R., Silva-Zuta M.Z., Maicelo-Quintana J.L., Cayo-Colca I.S. (2023). Conching process time, sauco by-product concentration, and sacha inchi oil levels identification for enrichment of dark chocolate. Heliyon.

[2] Barisci V., Kopjar M., Jozinovic A., Flanjak I., Ackar D., Milicevic B., Subaric D., Joick S., Babic J. (2019). The chemistry behind chocolate production. Molecules.

[3] Cahyani A., Kurniasari J., Nafingah R., Rahayoe S., Harmayani E., Saputro A.D. (2019). Determining casson yield value, casson viscosity and thixotropy of molten Chocolate using viscometer. IOP Conf. Ser. Earth Environ. Sci..

[4] Ertural Isik G., Gunes R., Said Tocker O., Palabiyik I., Konar N., Sagdic O. (2023). Importance of rheological properties in enrobing efficiency of dark chocolate: Application in wafer products. Int. J. Sci. Technol..

[5] Servais C., Ranc H., Roberts I.D. (2003). Determination of chocolate viscosity. J. Texture Stud.-Wiley Online Libr..

[6] Hendrik N.J., Marchesini F.H., Van de Walle D., Dewettinck K. (2023). Accurate evaluation of the flow properties of molten chocolate: Circumventing artefacts. Food Anal. Methods.

[7] Talansier E., Bacconnier A., Caton F., Chastel C., Costa L., Gunes D.Z., Roux D.C.D. (2019). Accurate methodology to determine slip velocity, yield stress and the constitutive relation for molten chocolate. J. Food Eng..

[8] Beckett S.T. (2017). Rheological properties. Beckett’s Industrial Chocolate Manufacture and Use.

[9] Guckenbiehl Y., Martin A., Ortner E., Rothkpf I., Schweiggert-Weisz U., Buettner A., Naumann-Gola S. (2022). Aroma-active volatiles and rheological characteristics of the plastic mass during conching of dark chocolate. Food Res. Int..

[10] Konar N., Palabiyik I., Karimidastjerd A., Said Toker O. (2024). Chocolate microstructure: A comprehensive review. Food Res. Int..

[11] Vásquez C., Henríquez G., López J.V., Penott-Chang E.K., Sandoval A.J., Müller A.J. (2019). The effect of composition on the rheological behavior of commercial chocolates. LWT.

[12] De Graef V., Depypere F., Minnaert M., Dewettinck K. (2019). Chocolate yield stress as measured by oscillatory rheology. Food Res. Int..

[13] Mewis J., Wagner N.J. (2009). Thixotropy. Adv. Colloid Interface Sci..

[14] Scholfield C.R. (1981). Composition of soybean lecithin—Scholfield. J. Am. Oil Chem. Soc.-Wiley Online Libr..

[15] Caparosa M.H., Hartel R.W. (2020). Characterizing Lecithin Interactions in Chocolate Using Interfacial Properties and Rheology. J. Am. Oil Chem. Soc..

[16] Bastida-Rodríguez J. (2013). The Food Additive Polyglycerol Polyricinoleate (E-476): Structure, Applications, and Production Methods. Int. Sch. Res. Not..

[17] Martinez-Padilla L.P. (2024). Rheology of liquid food under shear flow conditions: Recently used models. J. Texture Stud..

[18] Hussain N., Pei Er W., Rajentran K. (2024). Effects of polyglycerol polyricinoleate (PGPR) as partial substitute of coca butter and lecithin on rheology and shelf life of chocolate glaze. J. Biochem. Microbiol. Biotechnol..

[19] Prosapio V., Norton I.T. (2019). Development of fat-reduced chocolate by using water-in-cocoa butter emulsions. J. Food Eng..

[20] Afoakwa E.O., Paterson A., Fowler M. (2007). Factors influencing rheological and textural qualities in chocolate—A review. Trends Food Sci. Technol..

[21] Beckett S.T. (2018). The Science of chocolate. Royal Society Chemistry.

[22] Chevalley J. (1975). Rheology of chocolate. J. Texture Stud..

[23] Harris T.L. (1968). Surface Active Lipids in Foods.

[24] Van Nieuwenhuyzen W. (1997). Functionality of lecithins. Fett-Lipid.

[25] Wilson L. (1991). Yiled Stress Studies on Molten Chocolate. Ph.D. Tesis.

[26] Afoakwa E.O., Paterson P., nd Fowler M. (2008). Effects of particle size distribution and composition on rheological properties of dark chocolate. Eur. Food Res. Technol..

[27] Saputro A.D., Van de Walle D., Caiquo B.A., Hinneh M., Kluczykoff M., Dewettinck K. (2019). Rheological behaviour and microstructural properties of dark chocolate produced by combination of a ball mill and a liquefier device as small-scale chocolate production system. LWT.

[28] Bahari A., Akoh C.C. (2018). Texture, rheology and fat bloom study of ‘chocolates’ made from cocoa butter equivalent synthesized from illipe butter and palm mid-fraction. LWT.

[29] Glicerina V., Romani S., Ahmed E.J., Basu S. (2017). Chapter 24—Advances in yield stress measurements for chocolate. Advances in Food Rheology and Its Applications.

[30] Biswas N., Cheow Y.L., Tan C.P., Siow L.F. (2017). Physical, rheological and sensorial properties, and bloom formation of dark chocolate made with cocoa butter substitute (CBS). LWT-Food Sci. Technol..

[31] Dunnewind B., Janssen A.M., Van Vliet T., Weenen H. (2004). Relative importance of cohesion and adhesion for sensory stickiness of semisolid foods. J. Texture Stud..

